# Impaired differentiation potential of CD34-positive cells derived from mouse hair follicles after long-term culture

**DOI:** 10.1038/s41598-022-15354-9

**Published:** 2022-06-30

**Authors:** Yukiteru Ouji, Masayasu Misu, Tomotaka Kitamura, Daisuke Okuzaki, Masahide Yoshikawa

**Affiliations:** 1 Department of Pathogen, Infection and Immunity, Nara Medical University, 840 Shijo-cho, Kashihara, Nara 634-8521 Japan; 2 Genome Information Research Center, Research Institute for Microbial Diseases, Osaka University, Suita, Osaka, Japan

**Keywords:** Biological techniques, Medical research

## Abstract

Hair follicle epithelial stem cells (HFSCs), which exist in the bulge region, have important functions for homeostasis of skin as well as hair follicle morphogenesis. Although several methods for isolation of HFSCs using a variety of stem cell markers have been reported, few investigations regarding culture methods or techniques to yield long-term maintenance of HFSCs in vitro have been conducted. In the present study, we screened different types of commercially available culture medium for culturing HFSCs. Among those tested, one type was shown capable of supporting the expression of stem cell markers in cultured HFSCs. However, both the differentiation potential and in vivo hair follicle-inducing ability of HFSCs serially passaged using that optimal medium were found to be impaired, probably because of altered responsiveness to Wnt signaling. The changes noted in HFSCs subjected to a long-term culture suggested that the Wnt signaling-related environment must be finely controlled for maintenance of the cells.

## Introduction

Stem/precursor cells in skin tissue retain the homeostasis state of the epidermal layer, hair follicles, and sweat glands^1–3^. In particular, hair follicle stem cells (HFSCs), which are located at the base of the upper permanent portion of the follicular outer root sheath, have important roles in skin tissue regeneration and hair cycles, as well as hair morphogenesis^4–7^. HFSCs isolated from skin are considered to provide important information regarding hair biology, which is useful for stem cell technology related to tissue engineering including transplantation and organoid formation.

Previous studies have shown that HFSCs express several markers, including CD34, CD133, cytokeratin15 (CK15), and leucine rich repeat containing G protein-coupled receptor 5 (Lgr5)^8–12^. Although methods for obtaining HFSCs from mouse skin by use of those markers have been reported^13–18^, few investigations regarding culture methods or techniques to yield long-term maintenance of HFSCs in vitro have been conducted^19,20^. Barrandon et al*.* found that use of 3T3 fibroblast cells as feeder cells was helpful for long term cultures^21^, and others have reported that use of cytokines containing growth factors along with the extracellular matrix (ECM) allowed for expansion and maintenance of HFSCs without feeder cells^19,20^. We also previously reported isolation of HFSCs from adult murine skin tissue by fluorescent activated cell sorting (FACS) using CD34 and CD49f. (α6 integrin), selective surface markers, and then maintenance of HFSCs in vitro for a prolonged period (at least 150 days) using Wnt signal protein Wnt-3a and sequential FACS sorting^22^. However, the procedures detailed in those studies require complicated processes, such as preparation of feeder cells, addition of cytokines and matrix, and repeated FACS-based cell isolation. Therefore, it is considered that a more accessible long-term culture model for propagating HFSCs is necessary.

In the present study, simple cultures starting from HFSCs in vitro, in which commercially available culture media developed for stem cell maintenance were utilized without adding exogenous factors such as serum or feeder cells, were screened. Among those tested, one type of medium was found capable of supporting expression of CD34 in cultured HFSCs. Although use of optimal medium for serially passaged cultures increased the size of the CD34-positive cell fraction in cultured HFSCs, both the potential for differentiation and ability to induce hair follicles in vivo were found to be impaired. Furthermore, increased Wnt-7a production was observed in HFSCs cultured for a long term, which was likely related to loss of both differentiation potential and hair follicle-inducing activity.

## Results

### Characterization of HFSCs passaged with various media for short term

**Figure 1:**
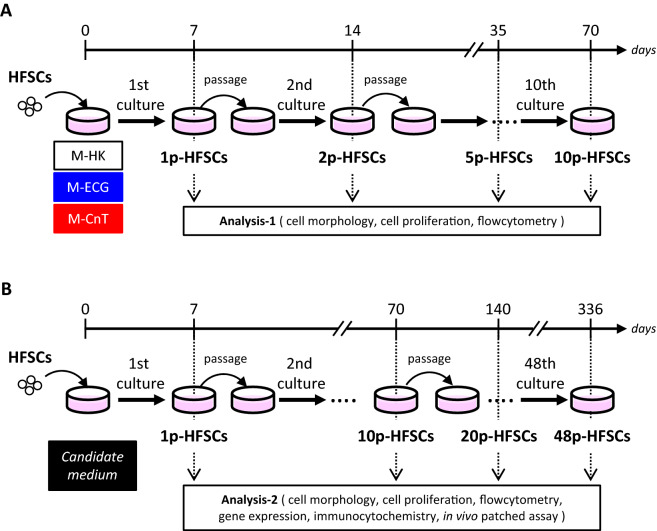
Analysis of short- and long-term cultured HFSCs. (**A**) HFSCs were cultured with various types of serum-free medium (M-HK, M-EcG, M-CnT) for a short term (10 passages), then a candidate medium was selected. (**B**) HFSCs were cultured with the candidate medium for a long term (48 passages) and subjected to various analyses.

**Figure 2:**
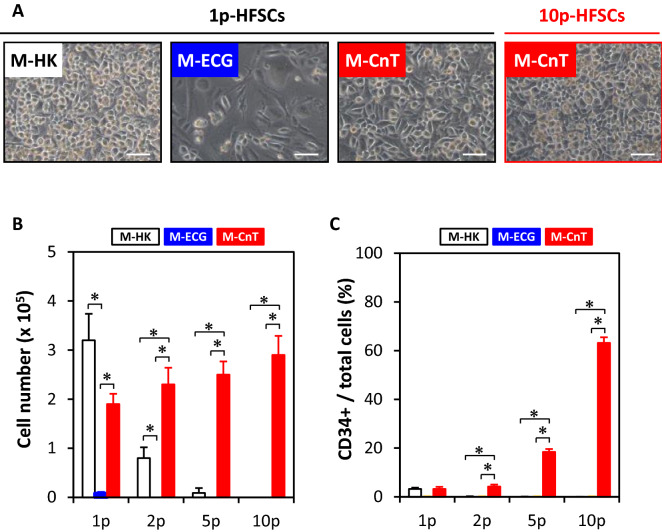
Characterization of HFSCs cultured in various medium types for short term. (**A**) Morphology results of primary HFSCs (1p-HFSCs) cultured with M-HK, M-ECG, or M-CnT for seven days. 10p-HFSCs denotes the morphology of HFSCs after 10 passages when cultured with M-CnT for 7 days. Scale bar = 50 mm. (**B**) Cell proliferation of HFSCs cultured with various types of medium (M-HK, M-ECG, M-CnT) for a short term (1p- to 10p-HFSCs). Assays of 2p-, 5p-, or 10p-HFSCs cultured with M-ECG, or 10p-HFSCs cultured with M-HK were not performed. *p < 0.05 (**C**) CD34-immunopositivity of HFSCs cultured with various types of medium (M-HK, M-ECG, M-CnT) for a short term (1p- to 10p-HFSCs). Assays of 2p-, 5p-, or 10p-HFSCs cultured with M-ECG, or 10p-HFSC cultured with M-HK were not performed. CD34-immunpositive cells were not detectable in 1p-HFSCs cultured with M-ECG, or 2p- and 5p-HFSCs cultured with M-HK. *p < 0.05.

First, HFSC morphology, proliferation, and CD34 expression were examined after separate culturing with three types of medium, i.e., M-HK, M-ECG, and M-CnT (Fig. 1A). Morphology findings of primary HFSCs (1p-HFSCs) cultured with M-HK or M-CnT for seven days showed similar cobblestone-like shapes (Fig. 2A; M-HK, M-CnT), while HFSCs cultured with M-ECG presented a different morphology, including a spindle-like shape shown for cells that had undergone differentiation (Fig. 2A; M-ECG). The proliferation ability of HFSCs cultured with M-CnT was maintained up to 10 passages (Fig. 2A,B; M-CnT), whereas that of HFSCs cultured with M-HK showed a decrease over serial passages and was completely lost by passage 10 (Fig. 2B; M-HK). On the other hand, HFSCs cultured with M-ECG completely lost ability to proliferate during the second culture (Fig. 2B; M-ECG). Interestingly, the CD34 immunopositivity of HFSCs cultured in M-CnT medium was increased at each examination up to 10 passages (1p-HFSCs, 3.2 ± 0.98%; 2p-HFSCs, 4.2 ± 0.87%; 5p-HFSCs, 18.4 ± 1.5%; 10p-HFSCs, 63.2 ± 3.1%) (Fig. 2C; M-CnT), whereas when the cells were cultured in M-HK or M-ECG, CD34-immunopositivity became undetectable by the second culture (Fig. 2C; M-HK, M-ECG). Therefore, M-CnT was selected as the medium for longer cultivation periods in the following experiments.

### Characterization of HFSCs passaged for long term in M-CnT medium

**Figure 3:**
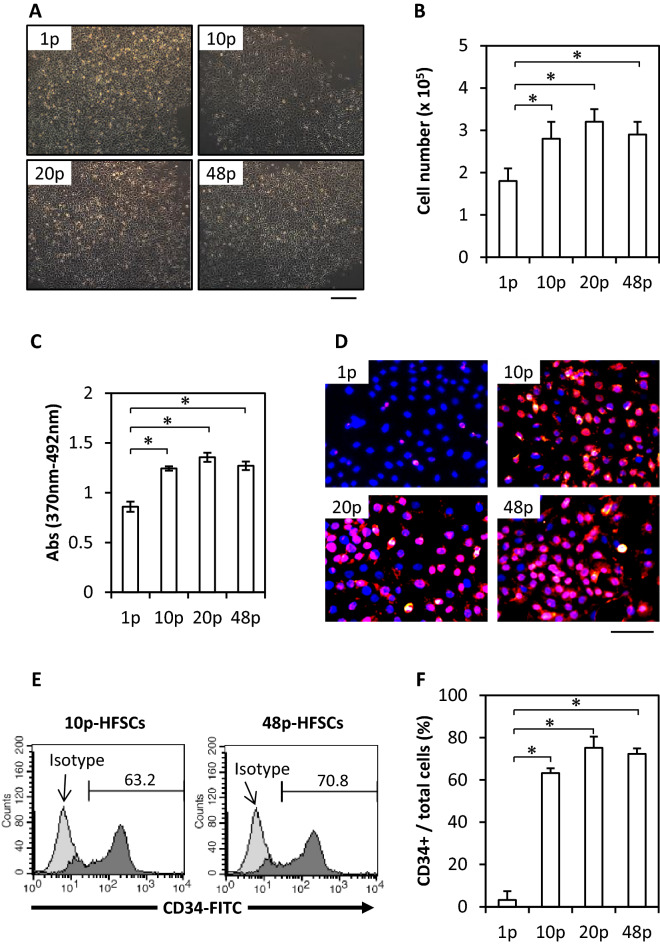
Characterization of HFSCs passaged with M-CnT for long term. (**A**) Morphology results of 1p-, 10p-, 20p-, and 48p-HFSCs cultured with M-CnT. Scale bar = 50 mm. (**B**) Cell proliferation of 1p-, 10p-, 20p-, and 48p-HFSCs cultured with M-CnT. *p < 0.05. (**C**) BrdU proliferation assay results of p1-, 10p-, 20p-, and 48p-HFSCs cultured with M-CnT. *p < 0.05. (**D**) Immunocytochemical results of CD34-positive cells among 1p-, 10p-, 20p-, and 48p-HFSCs cultured with M-CnT. CD34-positive (red) and nuclei (blue) were stained with an Alexa546 labeled secondary antibody and DAPI, respectively. Scale bar = 50 mm. (**E**) Flow cytometry results of 10p- and 48p-HFSCs cultured with M-CnT. (**F**) CD34-immunopositivity of HFSCs cultured with M-CnT for 48 passages. *p < 0.05.

Next, we investigated the characteristics of HFSCs passaged with M-CnT for a long term (Fig. 1B). Comparisons of 1p-, 10p-, 20p-, and 48p-HFSCs cultured with M-CnT showed no apparently different morphology findings (Fig. 3A), and proliferation ability was maintained or increased (Fig. 3B, C). Furthermore, the fraction of CD34-immunopositive cells gradually increased during the first 10 passages, with more than 70% of HFSCs shown to be CD34-immunopositive thereafter up to 48 passages (Fig. 3D–F). M-CnT, a commercially available serum-free culture medium, was found to maintain the morphology and proliferation ability of HFSCs along with a high rate of cells with CD34 expression during long-term cultures.

### Gene expressions of HFSCs passaged with M-CnT for long term

**Figure 4:**
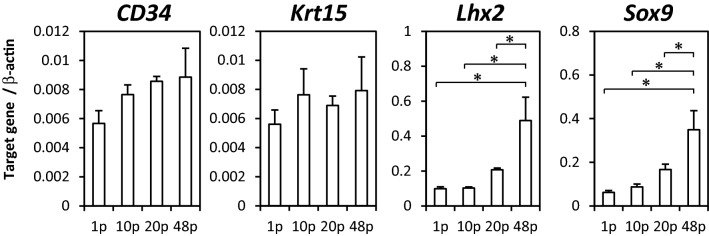
Gene expressions in HFSCs passaged with M-CnT for long term. Gene expressions of epithelial stem cell markers (*CD34, Krt15, Lhx2, Sox9*) in cultures of 1p-, 10p-, 20p-, and 48p-HFSCs with M-CnT were examined using real-time qRT-PCR. *p < 0.05.

Gene expressions of epithelial stem cell markers (*CD34, Krt15, Lhx2, Sox9*) were examined in HFSCs passaged with M-CnT using real-time qRT-PCR (Fig. 4). The expressions of *CD34* and *Krt15* were maintained up to 48 passages, while significant increases in *Lhx2* and *Sox9* were observed in 48p-HFSCs.

### HFSCs lost differentiation ability during long-term culture with M-CnT

**Figure 5:**
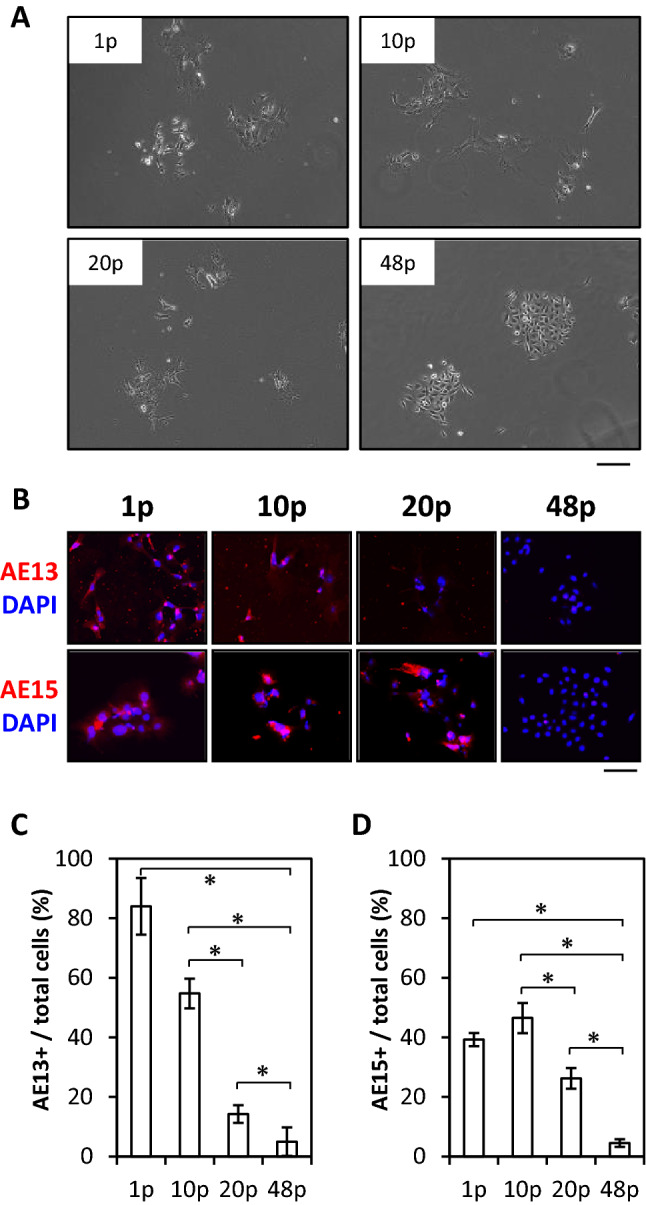
Lost differentiation ability of HFSCs during long-term culture with M-CnT. (**A**) Morphology results of 1p-, 10p-, 20p-, and 48p-HFSCs cultured with M-CnT and treated with Wnt-10b. Scale bar = 40 mm. (**B**) Immunocytochemical results obtained using AE13 and AE15, markers of cell differentiation, for 1p-, 10p-, 20p-, and 48p-HFSCs cultured with M-CnT and treated with Wnt-10b. Scale bar = 40 mm. (**C**), (**D**) Percentages of AE13- (**C**) and AE15-immunopositive (**D**) cells in 1p-, 10p-, 20p-, and 48p-HFSCs cultured with M-CnT. *p < 0.05.

The potential of HFSCs to differentiate into follicle epithelial cells was assessed by examining those passaged with M-CnT for a long term using Wnt-10b, which is known to promote differentiation of immature epithelial cells into HFSCs^23–25^ (Fig. 5). Significant morphological changes and inhibition of proliferation of 1p-, 10p- and 20p-HFSCs were induced by Wnt-10b, while those effects were limited in 48p-HFSCs (Fig. 5A). Moreover, immunocytochemical examinations of HFSCs were performed using AE13 and AE15, known markers of cell differentiation (Fig. 5B). AE13- and AE15-immunopositive cells were detected in 1p-, 10p- and 20p-HFSCs on M-CnT cultures with Wnt-10b treatment, whereas there were few or no immunopositive cells in cultures of 48p-HFSCs (Fig. 5C, D). These findings indicated that HFSCs cultured with M-CnT lost their differentiation ability over a long term.

### Hair follicle-inducing ability of HFSCs lost during long-term culture with M-CnT

**Figure 6:**
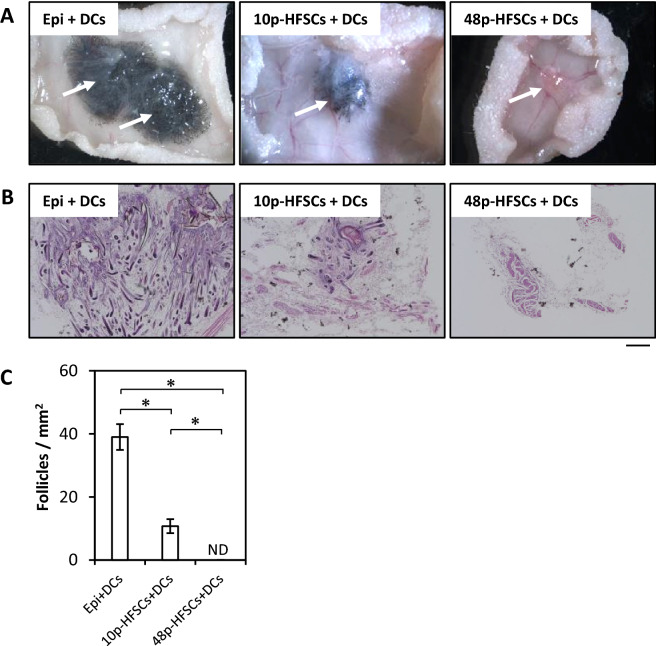
Hair follicle-inducing ability of HFSCs lost during long-term culture with M-CnT. (**A**) Results of in vivo patch assays using epidermal cells (Epi) as controls, 10p- and 48p-HFSCs passaged with M-CnT, and dermal cells (DCs) prepared from PD2 mice (Epi/DCs, 10p-HFSCs + DCs, 48p-HFSCs + DCs, respectively). (**B**) Analysis of reconstituted follicle tissues. Large numbers of hair follicles were detected in transplants of Epi or 10p-HFSCs cultured with M-CnT in combination with DCs (Epi/DCs, 10p-HFSCs + DCs, respectively), whereas that was not seen in 48p-HFSCs cultured with DCs (48p-HFSCs + DCs). Scale bar = 200 mm. (**C**) Number of mature hair follicles per mm^2^ in reconstituted skin. ND; not detectable. *p < 0.05.

To examine the potential of hair follicle formation of HFSCs cultured with M-CnT for a long term, in vivo patch assays were performed as a hair reconstitution experiment (Fig. 6). Epidermal cells (Epi) that included epithelial stem cells and dermal cells (DCs) were obtained from postnatal Day 2 mice, and used as the control. Large numbers of hairs were shown at four weeks after transplantation of Epi with DCs into nude mice (Fig. 6A, Epi + DCs), while a reduced level of hair induction, though still distinct, was observed using 10p-HFSCs in the reconstitution experiment (Fig. 6A, 10p-HFSCs + DCs). In contrast, there was no hair induction following transplantation of 48p-HFSCs (Fig. 6A, 48p-HFSCs + DCs). Transplantation of DCs, 10p-, or 48p-HFSCs alone also resulted in no hair induction (Supplementary Fig. S1). To examine formation of hair follicles in reconstituted skin, skin tissues were harvested, fixed, and sectioned, then stained with H&E for a histological examination (Fig. 6B). Large numbers of hair follicles were detected in transplants of Epi in combination with DCs (Fig. 6B, C, Epi + DCs), while a reduced level of hair follicle formation, though still distinct, was observed with transplantation of 10p-HFSCs with DCs (Fig. 6B, C, 10p-HFSCs + DCs). On the other hand, there was no follicle formation in tissues that received 48p-HFSCs (Fig. 6B, C, 48p-HFSCs + DCs). These results demonstrated that the ability of HFSCs to induce hair follicles was largely impaired or lost in cultures passaged for a long term.

### Changed responsiveness to Wnt signal inhibitors in HFSCs passaged with M-CnT during long-term culture

**Figure 7:**
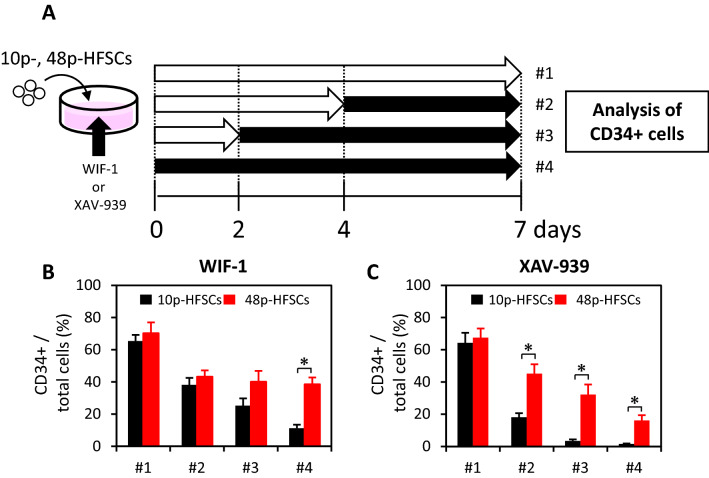
Changed responsiveness to Wnt signal inhibitors in HFSCs passaged with M-CnT for long term. (**A**) Schedules of experiments for determining responsiveness of HFSCs passaged with M-CnT for long term to Wnt signal inhibitors. Flow cytometry was used to examine CD34-immunopositivity in 10p- and 48p-HFSCs cultured with M-CnT after treatment without or with a Wnt signal inhibitor (WIF-1 or XAV-939) on Day 0, 2, and 4 (#4, #3, #2, #1, respectively). (**B**) Analysis of CD34-positive cells among 10p- and 48p-HFSCs cultured with WIF-1. *p < 0.05. (**C**) Analysis of CD34-positive cells among 10p- and 48p-HFSCs cultured with XAV-939. *p < 0.05.

Loss of differentiation and hair follicle-inducing abilities were observed in HFSCs passaged for a long term in M-CnT. Wnts are well known to be deeply engaged in maintaining the stemness of HFSCs^26–28^, and we previously reported that a CD34-positive population was dependent on Wnt-3a and its disappearance following addition of Wnt signal inhibitor to cultures^22^. Therefore, in the present study, the responsiveness of HFSCs to treatment with Wnt signal inhibitors (WIF-1, XAV-939) was examined (Fig. 7). WIF-1 or XAV-939 was added on Day 0, 2, and 4 of seven-day cultures of 10p- and 48p-HFSCs in M-CnT, and CD34-immunopositivity was examined using flow cytometry (Fig. 7A). In 10p-HFSCs, a remarkable reduction in number of CD34-immunopositive cells was observed following treatment with the signal inhibitors, while that reduction effect was limited in 48p-HFSCs (Fig. 7B, C). These results indicated a distinct difference in responsiveness to Wnt signal inhibitors between 10p-HFSCs and 48p-HFSCs.

### Altered characteristics of HFSCs after long-term passaged culture with M-CnT

**Figure 8:**
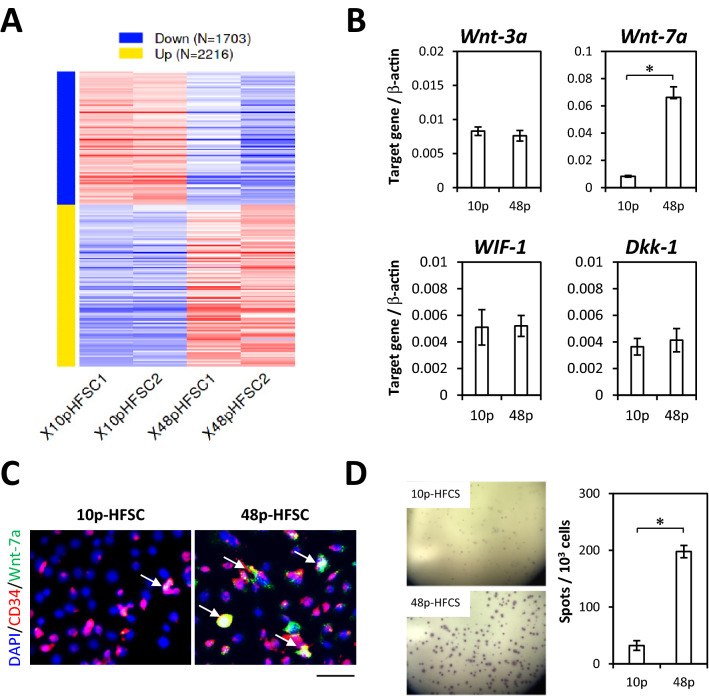
Altered characteristics of HFSCs after long-term passaged culture with M-CnT. (**A**) Heat map of significantly regulated genes in 10p- and 48p-HFSCs passaged with M-CnT during long-term culture produced using RNA-seq method. Data were visualized GO enrichment genes of differentially expressed genes (DEGs) extracted using the online application iDEP (ver. 0.951, http://bioinformatics.sdstate.edu/idep/). (**B**) Gene expression analysis of *Wnt-3a*, *Wnt-7a, Wif-1*, and *Dkk-1* in 10p- and 48p-HFSCs using qRT-PCR. *p < 0.05. (**C**) Immunocytochemical analysis of CD34 (red) and Wnt-7a (green) in 10p- and 48p-HFSCs passaged with M-CnT during long-term culture. Arrows indicate CD34- and Wnt-7a-double immunopositive cells. Scale bar = 50 mm. (**D**) ELISPOT assay of Wnt-7a in 10p- and 48p-HFSCs. *p < 0.05.

Regulatory gene expression related to cellular functions, including proliferation and differentiation, of 10p- and 48p-HFSCs passaged with M-CnT was determined using an RNA-seq method (Fig. 8A). The levels of expression of 2216 genes were found to be significantly increased in the 48p-HFSCs as compared to the 10p-HFSCs, while those of 1703 genes were decreased (Supplementary Fig. S2A−S2D). The top 20 upregulated genes among differentially expressed genes (DEGs) are shown in Table S2, most of which belonged to pathways that included signaling receptor activator and cytokine activities, signaling receptor binding, and other similar factors (Supplementary Fig. S2E, Supplementary Table S3). On the other hand, gene expressions in signaling pathways known to have influence on maintenance of HFSCs, such as TGF-b, BMP, or Shh, were not significantly changed. Notably, some Wnt ligands, including *Wnt-4, Wnt-5a, Wnt-7a, Wnt-7b, Wnt-9a*, and *Wnt-10a*, were found to be up-regulated in 48p-HFSCs, with a prominent increase in *Wnt-7a* noted (Supplementary Table S2). Our previous study demonstrated that CD34-positive cells produced Wnt-3a protein, while CD34-negative cells secreted Wif-1 and Dkk-1 proteins as Wnt inhibitors^22^. Therefore, the gene expression of *Wnt-7a* as well as *Wnt-3a*, *Wif-1*, and *Dkk-1* in 10p- and 48p-HFSCs was examined using qRT-PCR. As expected, based on the results of RNA-seq analysis, *Wnt-7a* expression showed a remarkable increase in 48p-HFSCs as compared to 10p-HFSCs, while such an increase was not seen with *Wnt-3a, Wif-1*, or *Dkk-1* (Fig. 8B). Moreover, immunocytochemical results confirmed that Wnt-7a-producing cells were CD34 positive (Fig. 8C) and that nearly all Wnt-7a-immunopositive cells were restricted to the CD34-positive population (Supplementary Fig. S3). Furthermore, ELISPOT assay findings showed a significantly greater number of Wnt-7a-secreting cells among 48p-HFSCs as compared to 10p-HFSCs (Fig. 8D), suggesting that the Wnt-7a production activity of HFSCs acquired during long-term passaged cultures with M-CnT may lead to an altered responsiveness to Wif-1 and Dkk-1, as observed in comparisons between 10p- and 48p-HFSCs (Fig. 7B,C).

## Discussion

Hair follicle stem cells (HFSCs) are localized in the bulge region, and important for maintaining homeostasis and differentiation of follicle epithelial cells, as well as hair morphogenesis^29–31^. Although a method for isolation of HFSCs using CD34/CD49f.-double immunopositivity as well as the characteristics of these cells have been reported^9,11^, few studies have investigated effective culture methods or techniques to yield long-term maintenance of HFSCs in vitro^19–21^. In the present study, three different types of commercially available culture medium were screened by culturing HFSCs without exogenous factors for a short term (up to 10 passages). Among the three tested media, only M-CnT produced a CD34-positive cell population in passaged cultures, with the ratio of that population increased up to 60% by passage 10 and then maintained at a similarly high level up to passage 48.

Based on our prior results as well as those noted in other studies, we considered that it is very difficult or nearly impossible to maintain cultures of HFSCs. In our previous study, CD34-positive cells disappeared within a few days of culture^22^, though addition of Wnt-3a delayed their disappearance. On the other hand, studies that used cytokines, ECM preparations, and bioengineering technology have reported high percentages of CD34-positive cells in cultures. Takeo et al*.* found that mixed additions of various cytokines/growth factors in cultures with collagen matrix brought about a CD34-positivity rate of 96% in mouse HFSC culture experiments^19^. Chen et al*.* also noted a high percentage (> 90%) of CD34-positivity in cultures of mouse or human HFSCs by use of a nanoscale biomimetic ECM composed of gelatin and alginate^32,33^. However, HFSCs passaged for a long term were not used in any of those studies, thus continuous maintenance of HFSCs at a high percentage remains a problem to be solved. In the present study, cultures that used M-CnT alone, a commercially available base medium, without additional reagents or matrix added showed an ability to maintain HFSCs at a relatively high percentage, greater than 60%, up to the 10th passage. Thus, we consider that this simple culture method is useful for selection and concentration of CD34-positive HFSCs cells. Although the complete composition of M-CnT has not been disclosed, the manual from the manufacturer states that the medium contains FGF proteins. Interestingly, FGF proteins were also reported to be contained in cytokine cocktails for original media established by Takeo et al*.*^19^ and Chacon-Martinez et al*.*^20^, suggesting that FGF signaling has an important role in maintenance of HFSCs.

That study presented by Chacon-Martinez et al*.*^20^ demonstrated the importance of a population balance between CD34-positive and -negative cells to provide a suitable microenvironment for HFSCs, which was also noted in our own study^22^, indicating that stem cells can be retained with a stable equilibrium of bidirectional interconversion between CD34-positive and -negative cells in such an environment, along with other influential factors^34,35^. The increased CD34-positive cell population observed in passaged cultures using M-CnT for a long term in the present study might have disrupted the finely controlled balance between CD34-positive and -negative cells, leading to loss of ability for differentiation and HF-induction of HFSCs.

Other reports have noted that CD133 is a marker of tumor-initiating cells (TICs)^36,37^ as well as HFSCs^10,38^. Although tumor generation was not observed in the present mice after transplantation with HFSCs, we examined CD133 gene expression in HFSCs cultured with M-CnT using real-time qRT-PCR (Supplementary Fig. S4). Those findings indicated that the expression of CD133 did not change in HFSCs passaged with M-CnT, suggesting that they had not shifted into TICs.

We also investigated the potential of HFSCs cultured with M-CnT for a long term to differentiate into follicle cells and whether they possessed an ability to form hair follicles. Although both Wnt-3a and Wnt-10b are canonical Wnts, Wnt-10b promotes differentiation of HFSCs^23,24^, while Wnt-3a functions to maintain their undifferentiated state^22^. AE13 and AE15, differentiated follicle cell markers, were detected in 1p- and 10p-HFSCs cultured with M-CnT in the presence of Wnt-10b, but not in 48p-HFSCs (Fig. 5). Along with the loss of differentiation potential as the number of passages increased, patch assay findings showed that HF-forming ability was also undetected or lost over time (Fig. 6). We considered that culturing of HFSCs with M-CnT for a long term might inhibit their potential for differentiation into mature follicle cells as well as their HF-inducing ability by epigenetic regulation of transcriptional factors, such as *Lhx2* and *Sox9*, because up-regulation of some transcription factor genes related to an undifferentiated state is known to be involved in establishment of induced pluripotent stem cells and directly reprogrammed cells^39–42^. Indeed, we observed significant increases in *Lhx2* and *Sox9* gene expression in 48p-HFSCs (Fig. 4), though additional analysis of epigenetic gene regulation of HFSCs cultured with M-CnT is needed to elucidate the mechanism related to this inhibition of differentiation.

In our previous in vitro study that used sequential maintenance of HFSCs with FACS sorting, CD34-positive cells were found to secrete the Wnt-3a, while CD34-negative cells secreted Wnt inhibitors such as Wif1, Dkk1^22^. Therefore, the effects of inhibition of Wnt signaling in HFSCs cultured with M-CnT were investigated in the present investigation, which showed that Wnt inhibitors moderately decreased the population of CD34-positive cells in 10p-HFSCs, while that was decreased in a limited manner in 48p-HFSCs (Fig. 7B, C). Although the expressions of *Wnt-3a* and Wnt signal inhibitors, such as *Wif-1* and *Dkk-1*, did not change in HFSCs after culturing with M-CnT for a long term (Fig. 8B), RNA-seq analysis demonstrated that some Wnts and hair follicle-related genes, including *Krt17* and *Krt18*, were increased in 48p-HFSCs as compared to 10p-HFSCs (Supplementary Table S2 and S3). In those, Wnt-7a expression was remarkably increased (D). Wnt-7a signaling has been reported to be involved in follicle neogenesis^43,44^, and Wnt-7a has also been shown to induce Sox9 expression through activation of b-catenin^45^. The present results suggest that surplus production of Wnts beyond Wnt inhibitors leads to an increase in expression of epithelial stem cell-related genes, such as *Lhx2* and *Sox9*^46–49^, in HFSCs cultured with M-CnT for a long term. Additionally, we consider that the inability to induce differentiation as well as unsuccessful formation of hair follicles seen in our experiments with long-term cultured HFSCs with M-CnT was due to a disrupted balance of Wnt signaling.

## Materials and methods

### Reagents

Mouse Wnt-10b recombinant protein (R&D Systems Inc., Minneapolis, MN) was used in cell cultures at a final concentration of 50 ng/ml. Mouse WIF-1 recombinant protein (R&D Systems Inc.) and XAV-939 (Wako Pure Chemicals ,Osaka, Japan), Wnt signal inhibitors, were used in cell cultures at a final concentration of 1 mg/ml and 1 mM, respectively.

### Mice

Inbred 8-week-old female C3H/HeN and BALB/c nude (*nu/nu*) mice were purchased from Japan SLC (Hamamatsu, Japan), and housed in group cages at the animal facilities of Nara Medical University. Four-week-old adult and postnatal day (PD) 2 C3H/HeN mice were used for preparation of HFSCs and dermal fibroblast cells (DCs), respectively. BALB/c *nude* mice were utilized for in vivo patch assays. The animal experimental protocols were approved by The Animal Care and Use Committee at Nara Medical University (no. 12111). All animal experiments including the surgical steps were performed in accordance with the guidelines of Nara Medical University and Animal Research: Reporting of In Vivo Experiments (ARRIVE) guidelines.

### Preparation of HFSCs by FACS

Adult C3H/HeN mouse whole skin epithelial cells were isolated from dorsal skin areas by trypsin treatment, as previously reported^22^, then single cell suspensions in phosphate buffer were exposed to antibodies directly coupled with fluorochrome for 30 min on ice. Antibodies used for FACS analysis were anti-α6 integrin (CD49f.) or rat IgG2a isotype (control) directly coupled with PE (R&D Systems, Inc.), and anti-CD34 (Bio-Rad; formerly AbD Serotec, Hercules, CA) or rat IgG2a isotype (control) (eBioscience, San Diego, CA) coupled with FITC. After washing twice with phosphate buffer, cell purification was performed using a FACSAria system equipped with the FACS DiVa software package (BD Biosciences, San Jose, CA). HFSCs were gated for single events, then sorted according to the expression of CD49f. and CD34. The purity of sorted cells was determined by post-sort FACS analysis and typically exceeded 95%.

### Culture assay

HFSCs were collected from dorsal skin epithelial cell populations using FACS and suspended with commercially available serum-free culture media, including M-HK, Humedia-KG2 (Kurabo, Osaka, Japan, https://www.kurabo.co.jp/bio/product/products.php?M = T&TID = 169); M-ECG, Epi Cell Growth Medium (Sigma, St. Louis, MO, https://www.sigmaaldrich.com/JP/ja/product/SIGMA/215-500); and M-CnT, CnT-PR (formerly CnT-07) (CELLnTEC, Bern, Switzerland, https://cellntec.com/products/cnt-pr/) at a density of 1 × 10^3^ cells per 35-mm dish, then cultured with one passage per week (Fig. 1). Culture medium was changed every two days. CD49f. + CD34 + cells isolated from dorsal skin epithelial cell populations were termed HFSCs and cells grown after the first culture of HFSCs were termed 1p-HFSCs, then 1p-HFSCs were collected and used for the second and successive cultures, with the same naming rule utilized for subsequent passaged cells.

For “Analysis-1”, cell morphology and proliferation, and CD34 expression were analyzed using 1p-, 2p-, 5p-, and 10p-HFSCs (Fig. 1A), then the most suitable medium for proliferation and maintenance of CD34 expression was selected. Next, for “Analysis-2”, HFSCs were further cultured using the selected medium for a long term, until the end of 48 passages, with 1p-, 10p-, 20p-, and 48p-HFSCs subjected to examinations, including gene expression, immunocytochemistry, and in vivo hair patch assays, in addition to those done in Analysis-1 (Fig. 1B).

### Cell proliferation assay

HFSCs or passaged HFSCs were plated in serum-free culture medium at a density of 1 × 10^3^ in 35-mm dishes, and cultured for seven days. Cell numbers were determined using the trypan blue exclusion method.

### BrdU proliferation assay

To examine BrdU proliferation, 1p-, 10p-, 20p-, and 48p-HFSCs were separately plated in serum-free culture medium at a density of 1 × 10^3^ in 96-well plates, and cultured for two days. Subsequently, BrdU (5-bromo-2′-deoxyuridine) was added and incubation was continued for an additional 24 h. BrdU incorporation was detected using a Cell Proliferation ELISA BrdU (colorimetric) kit (Roche, Sigma-Aldrich).

### Flow cytometry

HFSCs cultured in vitro were collected, then single cells were suspended in phosphate buffer and exposed to antibodies (CD34, CD49f., or isotype controls) for 30 min on ice. After washing twice with phosphate buffer, cells were gated for single events and CD49f.-immunopositive cells using a FACSCalibur (BD Biosciences). Analysis of CD34-immunopositive cells as an undifferentiated marker of HFSCs was then performed.

### Real-time qRT-PCR

Total RNA (1 µg) was extracted from cultured cells using TRIzol reagent (Invitrogen). Reverse transcription and qPCR were performed with a SYBR PrimeScript RT-PCR Kit II (TaKaRa), according to the manufacturer’s instructions, using primers purchased from TaKaRa Bio, Inc. (Supplementary Table S1). Target gene PCR product amounts were calculated relative to the internal control (β-actin), then compared between the experimental and control groups using the ΔΔCT method.

### In vitro differentiation of HFSCs

For examination of in vitro differentiation, 1p-, 10p-, 20p-, and 48p-HFSCs were separately plated in serum-free culture medium, with or without 50 ng/ml Wnt-10b, at a density of 1 × 10^3^ in 35-mm dishes and then cultured. After cultivation for seven days, observations of cell morphology, as well as immunocytochemical examinations using AE13 and AE15, known markers of cell differentiation, were performed.

### Immunocytochemistry

Immunofluorescence analysis was performed using a standard protocol. Briefly, cells were fixed in 4% paraformaldehyde, then cellular membranes were permeabilized with 0.1% Triton X-100 in PBS containing 1% BSA (TPBS). Detection of CD34- and Wnt-7a positive cells was performed using anti-CD34 (clone B-6) and anti-Wnt-7a (clone E-9) antibodies, respectively (each diluted 1:100) (Santa Cruz, Dallas, Texas). For detection of hair cortex and trichohyalin, the antibodies used were anti-AE13 and AE15, respectively (each diluted 1:50) (Abcam, Cambridge, UK). Following incubation overnight at 4°C and washing with TPBS three times, AlexaFluor546 conjugated anti-mouse secondary antibodies (diluted 1:200, Molecular Probes, Invitrogen) were used to detect primary antibodies. All nuclei were stained with DAPI (Dojindo, Kumamoto, Japan). After incubation for one hour at room temperature and washing with TPBS three times, fluorescence was detected with a fluorescence microscopic imaging system (BZ-X, Keyence, Osaka, Japan). Images obtained were modified using the Photoshop CS software package (Adobe Systems, San Jose, CA) to selectively detect intensely fluorescent cells.

### In vivo patch assays

For hair reconstitution examinations, in vivo patch assays were performed as previously described by O. Veraitch, et al.^50^, with minor modifications. HFSCs passaged for a short or long term (10p and 48p-HFSCs, respectively), or epidermal cells (Epi) prepared from PD 2 C3H/HeN mice (control) were subjected to the assay. Dermal cells (DCs) containing dermal papilla cells were obtained from PD 2 C3H/HeN mice. Appropriate cell combinations (epithelial cells: HFSCs or Epi and/or DCs; cell number of each 2 × 10^6^) were mixed, washed with PBS, and re-suspended in 100 μl of PBS, then subcutaneously injected into BALB/c nude (*nu/nu*) mice. At four weeks after transplantation, skin tissues were harvested, fixed with 4% PFA, and embedded in OCT compound. Sections were prepared using a cryostat and stained with hematoxylin–eosin (H&E), then the number of mature hair follicles per mm^2^ in reconstituted skin was counted.

### Assay for HFSC responsiveness to Wnt signaling

To investigate the responsiveness of HFSCs to Wnt signaling, 10p- and 48p-HFSCs were separately plated in serum-free culture medium, with or without 1 mg/ml WIF-1 or 1 mM XAV-939, at a density of 1 × 10^3^ in 35-mm dishes. Addition of the inhibitors was performed on day 0, 2, or 4 (Fig. 7A). After cultivation for seven days, CD34-immunopositive cells were detected by flow cytometry.

### RNA-seq

Total RNA was extracted with TRIzol reagent (Invitrogen), according to the manufacturer's protocol, while library preparation was performed based on the manufacturer’s instructions using a TruSeq stranded mRNA sample prep kit (Illumina, San Diego, CA). Sequencing was performed with an Illumina NovaSeq 6000 platform in 101 bp single-end mode, with sequenced reads mapped to the mouse reference genome sequences (mm10) using TopHat (ver. 2.1.1) in combination with Bowtie2 (ver. 2.3.5.1) and SAMtools (ver. 1.2). The number of fragments per kilobase of exon per million mapped fragments (FPKMs) was calculated using Cufflinks (ver. 2.2.1). Access to raw data related to this study was provided under Gene Expression Omnibus (GEO) accession number GSE201445. Data were analyzed and heat maps, MA and scatter plots, and pathway clusters were generated using the online application iDEP (ver. 0.951, http://bioinformatics.sdstate.edu/idep/).

### ELISPOT assays

ELISPOT assays were performed as previously reported^22^. Millicell membranes, on which cells were cultured with M-CnT for 12 h, were washed three times with PBST and blocked with 1% BSA for one hour at 37°C. After washing with PBST, the Millicell wells were pre-treated with a VECTASTAIN ABC kit (Vector Lab., Burlingame, CA) and 10% H_2_O_2_, then subsequently incubated overnight with an anti-Wnt-7a antibody (clone E-9, diluted 1:100, Santa Cruz) at 4 °C. After washing with PBS, the wells were incubated with a biotinylated anti-mouse IgG antibody (1:500; Santa Cruz Biotech) and exposed to a VECTASTAIN ABC kit. Spots were visualized by addition of True Blue™ (SeraCare Life Sciences, Inc.) as a substrate solution. The number of spots in each well was then counted using a stereo dissecting microscope.

### Statistical analysis

Data are expressed as the mean ± SD of three independent experiments. Statistical significance was tested using Student’s *t* or Tukey’s test using GraphPad Prism 8.4.3 (GraphPad Software, San Diego, CA). *P* values < 0.05 were considered to indicate statistical significance.

## Supplementary Information

Supplementary Information.

## Data Availability

RNA-seq raw data have been uploaded to Gene Expression Omnibus (GEO) with accession number GSE201445. The datasets used and/or analyzed during the current study are available from the corresponding author upon reasonable request.
