# Supplementary material for: Impaired differentiation potential of CD34-positive cells derived from mouse hair follicles after long-term culture
Source: Sci Rep. 2022 Jun 30;12:11011. doi: 10.1038/s41598-022-15354-9 (PMC9247072; doi:10.1038/s41598-022-15354-9)
Supplement: Supplementary file 1 — Supplementary Information. [file 41598_2022_15354_MOESM1_ESM.pdf]

Supplementary Information

Impaired differentiation potential of CD34-positive cells derived from mouse hair follicles after long-term culture

Yukiteru Ouji, Masayasu Misu, Tomotaka Kitamura, Daisuke Okuzaki, Masahide Yoshikawa

* Supplementary Figures (Fig. S1 – S4)
* Supplementary Tables (Table S1 — S3)

Supplementary Fig. S1.

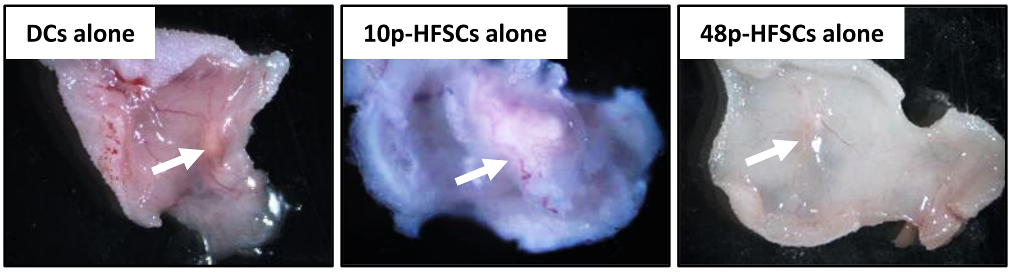

Images of skin tissues following transplantation with DCs or HFSCs
Transplantation of DCs, 10p-, or 48p-HFSCs alone also resulted in no hair induction. Arrows indicate transplantation points.

Supplementary Fig. S2.

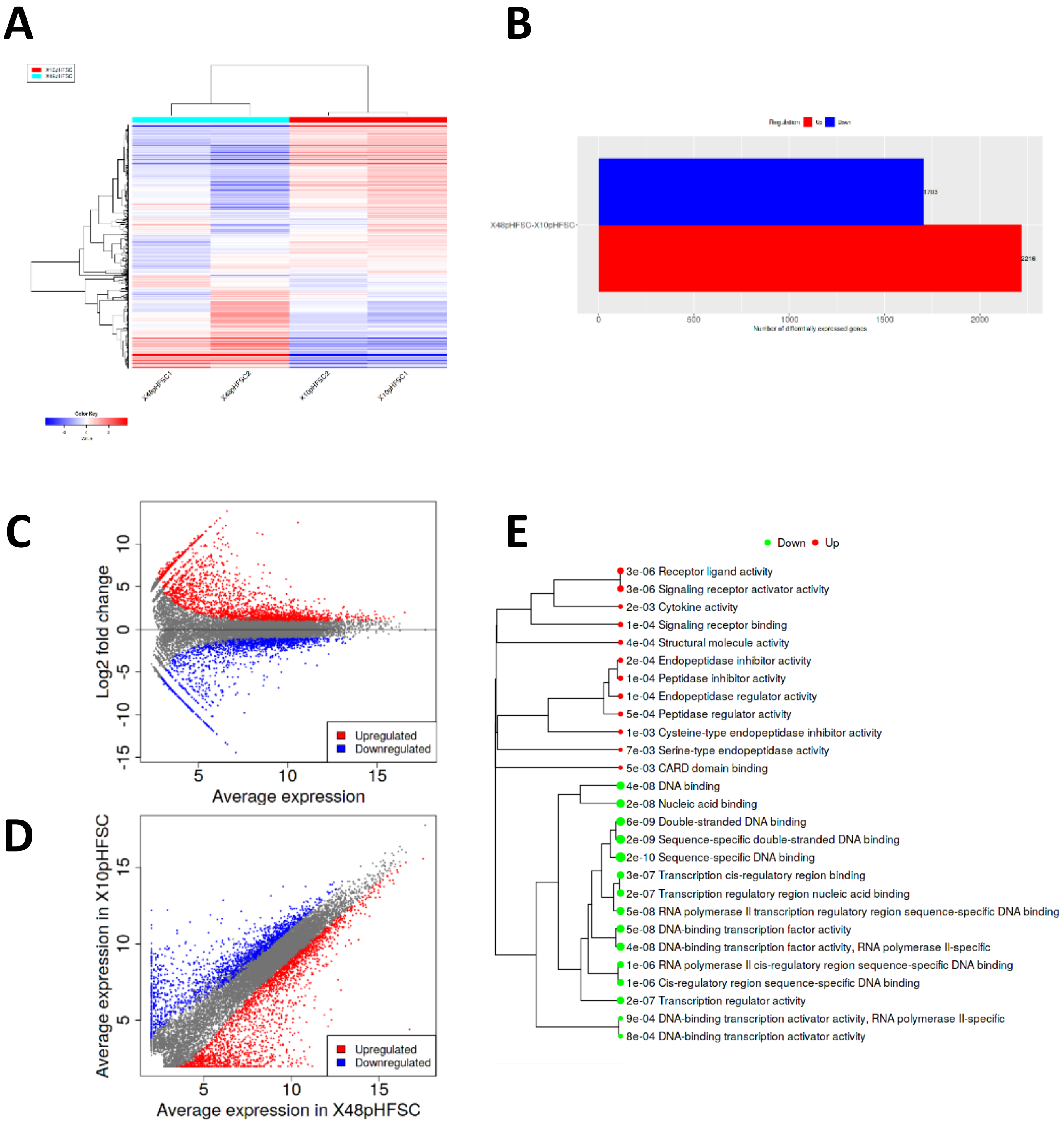

RNA-seq analysis for 10p- and 48-HFSCs

(A) Heat map visualized raw count data using online application iDEP (ver. 0.951, http://bioinformatics.sdstate.edu/idep/).

(B) Identified differential expressed genes (DEGs) were extracted using DESEq2. There were 2216 up-regulated and 1703 down-regulated genes.

(C, D) Significantly up or down-regulated genes were visualized by colored dots (red or blue, respectively) in MA plot (C) and scatter plot (D).

(E) Cluster of pathways enriched in DEGs.

Supplementary Fig. S3.

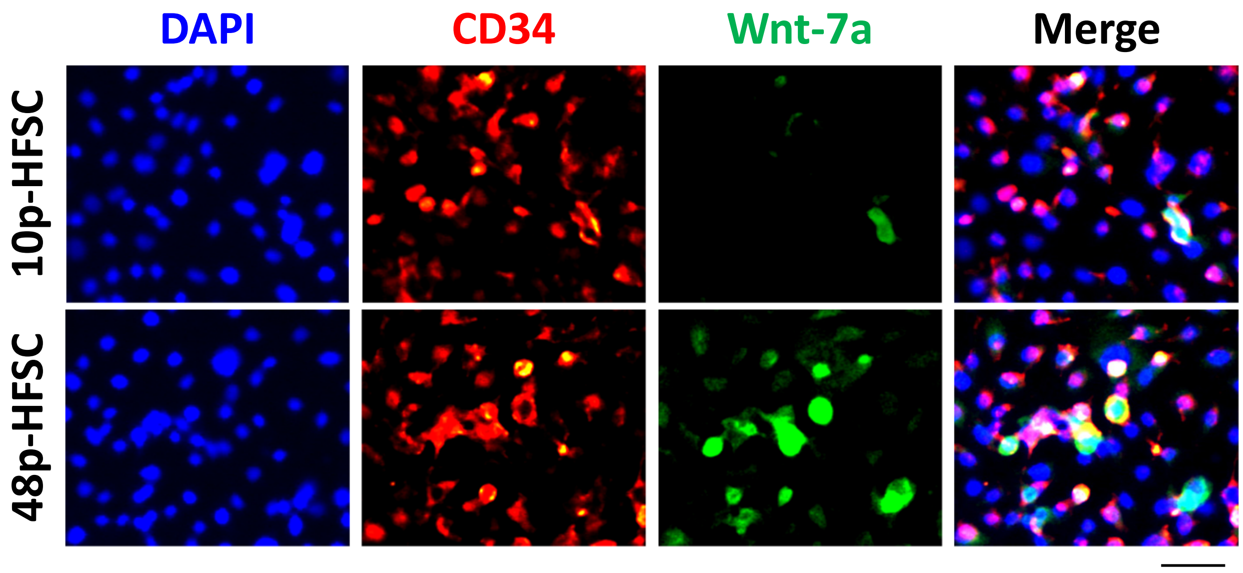

Immunocytochemical analysis of CD34 and Wnt-7a in 10p- and 48-HFSCs
Cells with Wnt-7a expression (green) and CD34-immunopositive cells (red) were detected. There was an increased in Wnt-7a-immunopositive cells among the 48p-HFSCs as compared to the 10p-HFSCs. Scale bar = 50 μm.

Supplementary Fig. S4.

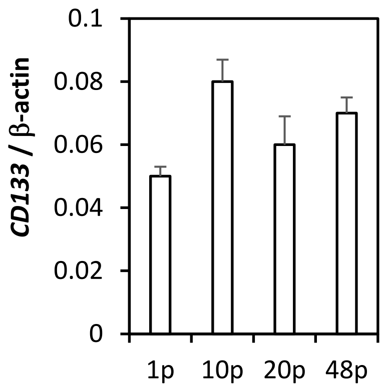

CD133 expression in HFSCs cultured with M-CnT for long term
CD133 gene expression in HFSCs cultured with M-CnT was examined using real time qRT-PCR. The level of CD133 expression did not remarkably change during the passaged cultures.

Supplementary Table S1

List of gene-specific primers used for real-time qRT-PCR

Gene	Forward primer seq.	Reverse primer seq.	Size of product (bp)	GeneBank accession no.
CD34	ACATGTGGTGGCTGACACAGAAC	CATGAGCTGAAGGCAGCATGA	107	NM_001111059.1
Krt15	CCGAGATTGGTACCAGAAGCAGA	GCGCGAGTTGTCAATGGTG	118	NM_008469.2
Lhx2	GGCAGATCTGGCAGCTTACAA	GCGCAAGCTGCTTCAAGTC	200	NM_010710.4
Sox9	GGACAACACATGCCTCTGCAA	TCTCCAGCCACAGCAGTGAGTAA	110	NM_011448.4
Wnt-3a	GGACACAGTCAAGCCGCAGA	CACTTCCAGGCCAGACAGACACTA	140	NM_009522.2
Wnt-7a	CGGACGCTCATGAACTTACACAA	GGTACAGGAGCCTGACACACCA	102	NM_009527.4
Wif-1	GCACGGCAGACACTGCAATAA	AAAGGCTGTGAACCCGGTGTAA	198	NM_011915.2
Dkk-1	CAGGTGTGCACCAAGCACAA	TCTGGCAGGTGTGGAGCCTA	142	NM_010051.3
CD133	TGTTAATGGCATTCTGTGTGGCTA	CCTCTGAATCCATCCTGCGATA	143	NM_001163577.1
β-actin	CATCCGTAAAGACCTCTATGCCAAC	ATGGAGCCACCGATCCACA	171	NM_007393.4

Supplementary Table S2

List of top 20 upregulated genes enriched in DEGs

Gene symbol	log2 fold change
Nxf7	13.86330445
Ap1m2	13.06909047
Pak3	12.7425168
Krt17	12.54095722
Dpep1	12.18389783
Entpd3	12.05993827
Rps6ka6	12.05296809
Dusp4	12.0116354
Tmem176a	11.85839324
Abcg2	11.5189237
Hmgcll1	11.50773172
Wnt7a	11.31055116
Krt18	11.31048329
Ralgps2	11.19839922
Rhox5	11.16325328
Nudt11	11.11327813
Tle4	11.08667591
Ifnar2	11.04498581
Trim30d	10.96309808
Pip5k1b	10.96218245

Supplementary Table S3

List of pathways enriched in DEGs

Direction	adj.Pval	nGenes	Pathways
Up-regulated	3.18E-06	64	Signaling receptor activator activity
	3.18E-06	64	Receptor ligand activity
	0.000149	182	Signaling receptor binding
	0.000149	36	Peptidase inhibitor activity
	0.000149	38	Endopeptidase regulator activity
	0.00017	35	Endopeptidase inhibitor activity
	0.000397	97	Structural molecule activity
	0.000501	42	Peptidase regulator activity
	0.001084	16	Cysteine-type endopeptidase inhibitor activity
	0.001706	30	Cytokine activity
	0.004838	7	CARD domain binding
	0.006736	28	Serine-type endopeptidase activity
Down-regulated	1.81E-10	206	Sequence-specific DNA binding
	1.66E-09	189	Sequence-specific double-stranded DNA binding
	5.78E-09	196	Double-stranded DNA binding
	1.83E-08	413	Nucleic acid binding
	3.58E-08	153	DNA-binding transcription factor activity, RNA polymerase II-specific
	3.58E-08	281	DNA binding
	4.72E-08	161	RNA polymerase II transcription regulatory region sequence-specific DNA binding
	4.72E-08	159	DNA-binding transcription factor activity
	2.03E-07	212	Transcription regulator activity
	2.32E-07	171	Transcription regulatory region nucleic acid binding
	2.61E-07	170	Transcription cis-regulatory region binding
	1.06E-06	138	Cis-regulatory region sequence-specific DNA binding
	1.28E-06	135	RNA polymerase II cis-regulatory region sequence-specific DNA binding
	0.000766	64	DNA-binding transcription activator activity
	0.000896	63	DNA-binding transcription activator activity, RNA polymerase II-specific

List of genes with signaling receptor activator activity enriched in DEGs

Wnt9a Pdgfb Sema4f Tgfb1 Jag2 Prl8a9 Ccl8 Csf2 Cxcl16 Ccl6 Sema6a Kitl Efemp1 Wnt5a Wnt7b Slurp1 Fam3b Tslp Sectm1a Il23a Pdgfa Wnt10a Jag1 Il1a Epha7 Sema3c Nppb Lrpap1 Cxcl5 Ppbp Cxcl3 Wnt7a Gas6 Vegfc Il15 Mif Sema3f Cxcl10 Ccl5 Wnt4 Apln Gdf15 Sema6c Metrnl Il18 Il7 Osgin2 Qrfp Cdc42ep2 Flrt2 Igf2 Pthlh Grem2 Lgals3 Insl6 Prl2c5 Uts2b Prl2c3 Prl7c1 Gmfg Prl2c1 Nrg1 Tnfsf18 Prl2c2

Log2 fold change: Wnt9a (2.1), Wnt5a (8.7), Wnt7b (1.2), Wnt10a (1.1), Wnt7a (11.3), Wnt4 (2.6)
